# The impact of the nurses’, doctors’ and clinical officer strikes on mortality in four health facilities in Kenya

**DOI:** 10.1186/s12913-020-05337-9

**Published:** 2020-05-26

**Authors:** Grace Kiringa Kaguthi, Videlis Nduba, Mary Beth Adam

**Affiliations:** 1 Centre for Respiratory Diseases Research-Kenya Medical Research Institute (KEMRI-CRDR), P.O. Box 62269-00200, Nairobi, Kenya; 2 AIC Kijabe Mission Hospital, Kijabe, Kenya

**Keywords:** Strikes, Mortality, Doctors, Nurses, Clinical officers, Universal health care, Kenya

## Abstract

**Background:**

Health worker strikes are a significant threat to universal access to care globally and especially in sub Saharan Africa. Kenya’s health sector has seen an increase in such industrial action. Globally there is limited data that has examined mortality related to such strikes in countries where emergency services were preserved. We sought to assess the mortality impact of an 100 day physician strike which was followed by 151 day nurses’ strike and 20 day clinical officer strike in Kenya.

**Methods:**

Monthly mortality data was abstracted from four public hospitals, Kenyatta National Referral Hospital, AIC Kijabe Hospital, Mbagathi Hospital and Siaya Hospital between December 2016 and March 2018. Differences in mortality were assessed using t-tests and multiple linear regression adjusting for facility, numbers of patients utilizing the hospital and department.

**Results:**

There was a significant decline in the numbers of patients seen, comparing the non-strike and strike periods; beta (ß) coefficient − 649 (95% CI -950, − 347) *p* < 0.0001. The physicians’ strike saw a significant decline in mortality (ß) coefficient − 19.0 (95%CI -29.2, − 8.87) *p* < 0.0001. Nurses and Clinical Officer strikes’ did not significantly impact mortality. There was no mortality increase in the post-strike period beta (ß) coefficient 7.42 (95%CI -16.7, 1.85) *p* = 0.12.

**Conclusion:**

Declines in facility-based mortality during strike months was noted when compared to a non-striking facility, where mortality increased. The decline is possibly associated with the reduced patient volumes, and a possible change in quality of care. Public health facilities are congested and over-utilized by the local population majority of whom cannot afford even low cost private care. Health worker strikes in Kenya where the public health system is the only financially accessible option for 80% of the population pose a significant threat to universal access to care. Judicious investment in the health infrastructure and staffing may decrease congestion and improve quality of care with attendant mortality decline.

## Background

The formal assessment of the impact of health workers’ strikes on mortality has demonstrated counterintuitive outcomes [1] contrary to mass media reports and reported health worker concerns [2, 3]. The nurses’ and doctors’ strikes in Kenya generated perceptions of unmet health needs [3]. Paradoxically, studies in industrialized nations show mortality to remain the same or decline [1] under strike conditions.

Data from a different, low resourced context has been limited [4]. Poor and disadvantaged persons report higher unmet needs for health care when there are strikes [5]. Suspension of health services may have unknown effects based on the organization of the health systems in that setting, health seeking behavior, morbidity patterns and the cadre of health professionals on strike as well as level of participation.

Health systems in developing countries are subject to a myriad of challenges including drug and vaccine stock-outs, understaffing and inadequate funding [6]. They’re sometimes skewed to curative rather preventive services. The predominant cadre of health workers is unique in different countries. In Kenya, in almost every case, there are roughly two Clinical officers (COs) for every doctor [7]. COs are similar to physician associates or physician assistants. They are medically trained over a period of 3 years in contrast to physicians whose basic training is at least for 5 years. They’re licensed to make medical decisions and are frequently the first point of contact, not the Medical Officer (MO). They retain the discretion to involve a physician in case management [8]. Contrarily, at the nation’s largest teaching and referral hospital, the Kenyatta National Hospital (KNH) there are two MOs for every CO [7]. Similarly, there were 1092 General Practitioners, 3208 COs, 20,371 nurses, 419 specialist MOs. This suggests the force of the doctors’ strike would be more perceptible in facilities without clinical officers.

The impacts of the strike in such a context are also influenced by health seeking behavior. Delays in seeking care have been documented for a range of illnesses not limited to tuberculosis [9, 10], malignancies [11, 12], pediatric care [13], maternal health and antenatal care [14]. Further fragility is introduced due to the difficult-to-quantify effects of endemic poverty. It undermines nutritional status and affects how people prioritize health. The majority of patients (80%) are not covered by health insurance [15]. They may not be able to afford care in private health facilities if government facilities were not fully operational. It has also been observed in developed nations, that a continuity in provision of emergency services [1, 16], could avert deaths and reduce the mortality impact of health worker strikes. However, the partial or complete disruption of such services in developing countries or a combination of all these factors could possibly aggravate effects of a health worker strike in developing versus developed nations [17].

### Kenya’s health system

To underpin this study, it’s useful to examine Kenya’s existing health system, infrastructure and utilization. According to the Kenya Master Health Facility List, only about 38% of Kenyan health facilities are managed by the private sector. Two of the largest public hospitals in this study are located in Nairobi where there is the largest concentration of private health facilities. That said, a considerable segment of the Kenyan population are unable to afford private sector health services and are therefore dependent on public hospitals. Reducing cost barriers to health services increased utilization of government health services demonstrated national scale changes to financing in maternity care. On June 1st 2013, government abolished user fees in all public dispensaries, health centers and all maternal health care including deliveries in all hospitals, compounding their over-utilization. Absent equivalent augmentation of facilities and personnel, this resulted in congestion, stock-outs and equipment break-down in said facilities [18, 19].

In addition, there were substantial data demonstrating severe staff shortages in public facilities [20]. Upon the promulgation of the new constitution in 2010, health care administration was decentralized and predominantly provided by county governments. Consequently, there was industrial action to protest this and a mass exodus of health workers, citing dilapidated health infrastructure, and underutilization of their skills.

Upstream, the strike may impact mortality and quality of care in alternative facilities. A not-for-profit hospital offering health care at a subsidized cost, recently reported increased patient volumes that exceeded the hospital’s ability to respond to needs over the period of the physician strike [4].

Finally, it is emergent that not all health personnel are created equal. A twenty-year study in New York State, an industrial action hotbed, showed nurses’ strikes to increase in-patient mortality and re-admission rates even after controlling for treatment intensity, disease severity and patient demographics [21]. However, there are also studies that detected no deleterious effects of nurses strikes [22, 23].

Given the variability in health systems, personnel funding, health seeking behavior, we sought to determine the impacts of the different health worker strikes in the context of a developing country, Kenya, with a view to understanding the contribution of each health worker to mortality outcomes.

The doctors’ strike was occasioned by failure of government to implement an agreement signed in 2013 to improve remuneration, staffing and equipping of health facilities and research funding. There was a high level of participation as all doctors employed in government facilities withdrew their services. Senior doctors (consultants) and military doctors were deployed to the national referral and teaching hospital. Services were restricted to emergencies; elective procedures were deferred or asked to seek assistance in private hospitals. In peripheral government hospitals, clinical officers who often act as a filter [8], took over operations and cases that they could not manage were possibly unattended to particularly if they couldn’t afford to pay for their transport to and management at private facilities. The nurses’ strike agitated for better pay and implementation of their previously agreed on collective bargaining agreement. The level of curtailment of care for all the health worker strikes was nearly complete except for Kenyatta National Hospital (KNH) whose nurses did not participate with their colleagues. The KNH is a parastatal with different employment terms.

## Methods

### Settings

We selected four hospitals that represent a spectrum of health facilities available in Kenya [1]; Kenyatta National referral hospital (KNH), which operates as a parastatal organization and whose nurses were not on strike during the 151 day nursing strike, [2] a private mission hospital, Kijabe Hospital (AICKH) whose health workers did not participate in any of the national unionized health worker industrial action, [3] a level 4 urban hospital (Mbagathi Hospital-MH) and, [4] a level 4 rural hospital (Siaya Hospital-SH).

### Timeline of the strikes in Kenya: duration and level of participation

The doctors’ industrial action lasted 100 days. At the inception of the doctors’ strike, nurses, too were called to strike, however they returned to work after a few days after reaching an agreement with county governments. They recommenced the strike on June 5, 2017, and terminated it 5 months later in November 2017. In the midst of the nurses’ strike, Clinical officers went on strike for 20 days. It has also been reported that the nurses’ at AICKH went on strike for 1 week in February in the midst of the doctors’ strike.

### Organization and selection of health facilities

We sampled three public and one private faith led facility. All were purposively selected to include a mix of high and medium to low patient throughput and level of specialization. The latter was selected to assess for upstream effects of the strike. Geographically, two were located within Nairobi, the country’s capital, one was 430 km (km) away from the capital in Western Kenya. The faith led facility was 60 km from Nairobi. Hospitals in the country are organized into levels one to six in ascending order based on complexity of services offered and the regional catchment it is intended to serve.

### Level 6 public facility (Kenyatta National Hospital-KNH)

This is the largest national referral hospital, with a 1455 bed capacity. It serves the largest catchment area, and is a teaching hospital for the University of Nairobi, the first in the country. It’s considered a tertiary facility by the ministry of health. All patients are managed by medical doctors from triage to admission and clinical officers are not involved in giving care. KNH nurses are employed by the national government under different terms and continued to work during the nurses’ strike.

### Level 6 private facility (AIC Kijabe Hospital-AICKH)

Located 1 hr’s drive from Nairobi, it has a 363 bed capacity, employs 600 staff. It is listed as a secondary care hospital by the ministry of health. It also is a teaching facility for nurses and post-graduate doctors. Patients are triaged by clinical officers and managed by both medical and clinical officers after admission.

### Level 4a public facility (Mbagathi Hospital-MH)

Considered a primary care facility, it’s located within the city’s capital, with a total bed capacity of 200 and staff of 328. Patients are triaged at the outpatient by medical doctors and managed thereafter by consultant physicians. Clinical officers have a nominal role.

### Level 4b public facility (Siaya Hospital-SH)

This is considered a secondary care hospital, located in rural western Kenya. It was included to increase the sample diversity and generalizability of results, has a lower patient throughput and a comparably lower level of specialization of services. Patients are triaged and largely managed by clinical officers, but medical officers are available to provide care for in patients. We could not obtain accurate information on bed count and staffing.

### Study design

This was an ecological survey to quantify any change in patient services and mortality during; any health worker strike period, compared to a non-strike baseline period and a 4 month post-strike period. Individual level data on diagnosis, demographics, access to care were omitted by design.

### Data collection

Data on deaths and numbers of patients seen was extracted in month to month time frames, consistent with government reporting standards from December 2015 to March 2018. Data was obtained from the following departments: outpatient, casualty, maternity/neonatal, HIV Comprehensive Care Clinic/Anti-Retroviral Treatment Clinic (ART), surgical and medical wards. Data was aggregated as needed for specific analysis. A comparison of average monthly outpatient visits, inpatient admissions and average monthly mortality rates during the 12 pre-strike months, the strike months, and post-strike months. The strike periods were as follows; (December 2016 to March 2017) for medical doctors, (June 2017 to November 2017) for nursing officers, (September 2017 to October 2017) for clinical officers. The baseline period was defined as the year preceding the strike (December 2015 to November 2016). The post-strike period was between December 2017 to March 2018, when health services were fully operational (no strikes in any cadre). These months were chosen since data are collected on a calendar-month basis. While the physician strike officially began December 5th 2016, an unofficial “go slow” mandate was in place within the public sector resulting in transfers from other facilities prior to, and in anticipation of, the formal walkout. The physician strike officially continued through March 14, 2017; however, we include the entirety of March as a strike month since all government physicians did not immediately return to work and patients remained uncertain of government service availability. The same is true of the CO strike which took place for 20 days, however the data was analysed over a 30 day month period.

Data from each department in each hospital month by month was entered on paper questionnaires and transcribed onto a Microsoft Access Database and analysed on Stata 13. (StataCorp, California US).

### Statistical methods

Frequency methods were used to sum up the number of patients seen and deaths per department in each facility in the baseline period. Independent t-tests was performed to compare the mean number of patients seen per facility and mean numbers of mortalities using the private level 6 hospital as the reference, during the strike and baseline periods. AICKH was the reference facility as it was the only private facility sampled comparable to the public hospitals. Furthermore, because patient demand overwhelmed its ability during the strike period, it was evidently the next level of financially accessible health care services for thousands during the strike [4], hence a suitable benchmark for the included public facilities. Linear regression was performed to compare total deaths in the baseline, strike and post-strike periods controlling for the numbers of patients seen, facility, individual departments at the hospitals and the caliber of health worker on strike. It was also used to model the numbers of patients seen, adjusting for the period (strike vs non-strike), facility and department, to determine which factors significantly accounted for the change in volumes of patients attended to.

## Results

**Fig. 1:**
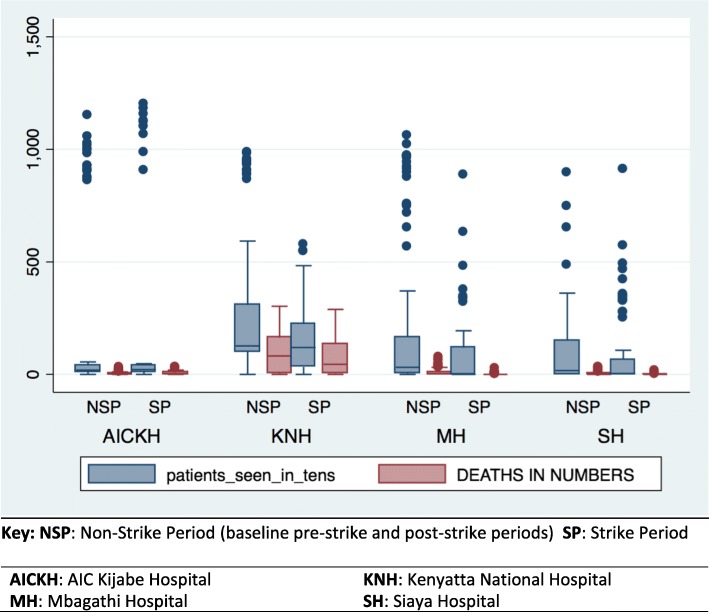
Box plot of patients seen (in tens) versus deaths, categorized by strike and non-strike periods

The total numbers of patients attended to in every hospital department were aggregated year to year for the study period. During the baseline period from December 2015 to November 2016, there were no significant differences in the mean numbers of patients seen between KNH and AICKH and MH. However, compared to AICKH the reference facility, SH saw significantly fewer patients (*p* = 0.02). In the 4 month post-strike period, KNH saw more patients than the reference facility, AICKH *p* = 0.008 (Table 1). Figure 1 shows the total number of patients seen versus deaths, by facility categorized by the strike or non-strike period. The non-strike period in this case aggregates the baseline pre-strike and post-strike periods.

**Table 1:** Total numbers of patients seen and respective deaths for the period under study

Facility	Dec 2015-Nov2016		Dec 2016-Nov 2017		Dec 2017-Mar 2018		T-test ***P*** value for differences in patients seen ***per year***		T-test ***p***-value for differences in ***all*** patients seen
	**patients**	**deaths**	**patients**	**deaths**	**patients**	**deaths**			
**AICKH**									
Casualty	113,390	44	119,897	48	9114	4	Dec2015-Nov2016	(Ref)	(Ref)
Maternity/neonatal	1368	20	2332	364	758	5	Dec2016-Nov2017		
Medical/Surgical	2614	212	2293	250	716	56	Dec 2017-Mar2018		
Pediatric casualty/in-patient	2107	66	1725	103	567	18			
ART clinic	5629	15	5142	10	1407	7			
**KNH**									
Casualty	111,243	1178	45,257	814	16,868	266	Dec2015-Nov2016	0.07	0.12
Maternity/neonatal	19,374	1822	15,873	1463	4551	400	Dec2016-Nov2017	0.55	
Medical/Surgical	28,264	4148	17,701	2973	7971	1203	Dec2017-Mar2018	0.008	
Pediatric casualty/in-patient	65,795	2911	42,478	2315	16,627	707			
ART Clinic	31,822	0	25,824	0	9743	0			
**MH**									
Casualty	108,134	72	56,911	43	16,428	28	Dec2015-Nov2016	0.88	0.38
Maternity/neonatal	7348	157	1962	33	2591	39	Dec2016-Nov2017	0.05	
Medical/Surgical	2893	522	603	110	701	122	Dec2017-Mar2018	0.26	
Pediatric casualty/in-patient	35,814	142	16,112	40	11,134	37			
ART clinic	18,435	16	15,600	10	4759	2			
**SH**									
Casualty	28,344	0	42,451	0	26,053	0	dec2015-nov2016:	0.03	0.02
Maternity/neonatal	3598	52	2457	350	1308	27	Dec2016-Nov2017	0.05	
Medical/Surgical	2383	247	755	64	592	32	Dec2017-Mar2018	0.14	
Pediatric casualty/in-patient	1333	55	104	16	224	6			
ART clinic	36,248	0	41,028		14,282	0

### Baseline period

At baseline (non-strike period), in-patients consistently had a significantly higher mortality in every facility. Mean outpatient deaths 14.0 (95%CI 7.31, 20.6) vs inpatients 41.6 (95%CI 32.0, 51.2) *p* = 0.0008. KNH had ten to twenty fold higher baseline mean mortality compared to all other facilities (Table 2).

**Table 2:** Mean baseline mortality Dec 2015-Nov 2016 (non-strike period) compared to reference hospital

Facility	Mean baseline mortality out-patients (95% CI)	Mean baseline mortality in-patients (95%CI)	***p***-value*	All Baseline Mortality (95% CI)	***p***-value^**#**^
AICKH	2.46 (1.38, 3.54)	6.34 (3.76, 8.92)	0.04	5.03 (3.25, 6.81)	ref
KNH	49.1 (27.6, 70.6)	123 (99.1, 148)	0.001	105 (85.0, 125)	< 0.0001
MH	3.83 (2.47, 5.19)	11.6 (7.56, 15.6)	0.03	9.60 (6.57, 12.8)	0.02
SH	0	6.00 (3.63, 8.37)	0.002	4.27 (2.50, 6.04)	0.55
All facilities mortality baseline	14.0 (7.31, 20.6)	41.6 (32.0, 51.2)	0.0008	41.5 (32.5, 50.4)	0.0001

### Strike period (Table 3)

Mean baseline out-patient mortality was higher than when any cadre of staff were on strike (Table 3). This was also true for in patient mortality. However, when any cadre of staff were on strike, in-patients had higher mean mortality 32.2 (95% CI 22.5, 41.8) vs outpatient 9.66 (95% CI 3.76, 15.6) *p* = 0.007. Nevertheless, this was still lower than the baseline period 41.6 (95%CI 32.0, 51.2).

**Table 3:** Baseline mortality versus mortality when different health workers are on strike in all facilities

Health Worker on strike	Mean baseline (non-strike mortality) out-patient across all facilities	Strike period out- patient mortality mean deaths (95% CI)	Mean baseline (non-strike mortality) in patient across all facilities	Strike period in- patient mortality mean deaths (95% CI)	p-value^**a**^
Doctors	15.1 (8.43, 21.8)	6.32 (2.02, 10.6)	45.3 (35.3, 55.3)	19.4 (9.59, 29.2)	0.11
Nurses		12.1 (2.24, 22.0)		40.6 (26.0, 55.1)	0.03
Clinical Officers		8.20 (−8.17, 24.6)		44.8 (17.6, 72.1)	0.13
Combined time of all cadres on strike		9.66 (3.76, 15.6)		32.2 (22.5, 41.8)	0.007

^a^ significant differences between in and out patient mortality based on cadre on strike

Adjusting for the numbers of patients seen in each facility, there were significantly fewer patients seen during the strikes compared to baseline; beta (ß) coefficient − .003 (95% CI −.006, −.001) *p* = 0.003. Compared to the baseline period, the doctors’ strike saw the largest decline in mortalities, (ß) coefficient − 19.0 (95% CI -29.2, − 8.87) *p* < 0.0001. The CO and nurses’ strikes also saw a decline in mortality that was not statistically significant (Table 4).

**Table 4:** Multiple linear regression model adjusting for patient numbers, staff cadre on strike, strike period, department and facility

Variable	Category	Beta coefficient (95% CI)	***p***-value	R squared
Total number of patients seen	N/A	−.003(−.006, −.001)	0.003	41.6%
Period	Baseline (non-strike)	REF		
	Doctors’ strike	−19.0 (−29.2, −8.87)	< 0.0001	
	Nurses’ strike	−3.27 (−14.7, 8.05)	0.57	
	Clinical Officers’ strike	−0.50 (−13.9, 12.9)	0.94	
	Post-strike	−7.42 (−16.7, 1.85)	0.12	
Department	Casualty	REF		
	Maternity/neonatal	−23.0 (−41.0, −5.08)	0.01	
	Medical/Surgical	6.71 (−11.4, 25.0)	0.47	
	Pediatric in and out-patient	−11.4 (−28.3, 5.49)	0.19	
	ART Clinic	−37.6 (−54.1, −21.0)	< 0.0001	
Facility	AICKH	REF		
	KNH	82.0 (72.0, 92.1)	< 0.0001	
	MH	−5.58 (−15.5, 4.33)	0.27	
	SH	−7.54 (−17.6, 2.57)	0.14	

Compared to the reference department (casualty/outpatient), maternity and the ART clinics had significant declines in mortality during the strike compared to the baseline period. While mortality at KNH declined in the strike period compared to baseline, the facility still had the highest mortality of all.

There was a statistically significant decline in the numbers of patients utilizing the health facilities when doctors beta (ß) coefficient − 649 (95% CI -951, − 347) *p* < 0.0001 and nurses were on strike beta (ß) coefficient,-353 (95% CI -692, − 13.8) *p* = 0.04, also in the post-strike period beta (ß) coefficient, − 435 (95%CI -712, − 158) *p* = 0.002, compared to baseline, but not when COs were on strike. However, the latter covered a period of 4 months only. All departments we sampled saw a decline in the numbers of patients seen, compared to casualty (Table 5).

**Table 5:** Determinants of the numbers of patients seen adjusted for: facility, department, strike period

Variable	Category	Beta coefficient (95% CI)	***p***-value	R squared
Period	Baseline (non-strike)	REF		66.4%
	Doctors’ strike	−649 (−951, −347)	< 0.0001	
	Nurses’ strike	−353 (−692, −13.8)	0.04	
	Clinical Officers’ strike	− 239 (− 641, 162)	0.24	
	Post-strike	−435 (−712, −158)	0.002	
Department	Casualty	REF		
	Maternity	− 6085 (− 6422, − 5748)	< 0.0001	
	Medical/Surgical	− 6034 (− 6384, − 5684)	< 0.0001	
	Pediatric in and out-patient	− 5382 (− 5728, − 5036)	< 0.0001	
	ART Clinic	− 4453 (− 4842, − 4063)	< 0.0001	
Facility	AICKH	REF		
	KNH	715 (417, 1013)	< 0.0001	
	MH	−3.19 (− 301, 295)	0.98	
	SH	− 485 (− 787, − 183)	0.002	

### Post-strike

There were no significant increases in mortality in the immediate period after the strike compared to baseline period beta (ß) coefficient-7.42 (95%CI -16.7, 1.85) *p* = 0.12.

## Discussion

There was a statistically significant decline in numbers of patients seen during the doctors’ strike and nurses’ strike but not during the CO strike period. This decline in patient numbers continued into the post-strike period. It is possible that even 3 months after the strikes had ceased patients were skeptical that services were fully available. After adjusting for the decline in patient volume, there was still a significant decline in mortality during the same period. Nurses and CO strikes did not significantly impact mortality in this [24] assessment; therefore the main focus of our deliberation is the doctors’ strike. During the doctors’ strike examination of departmental level data showed that, maternity and neonatal departments had a significant decline in mortality. KNH had the highest mortality overall at baseline and during the strike periods, consistent with its role as a very high acuity national referral hospital. The extent to which different unionized cadres respected the strike lines for their other health care workers is not data that is available but is partially represented in the numbers of patients seen during the respective time periods.

Maternity/neonatal department had significantly lower mortality during the strike period. This could be explained by lower numbers of high obstetric risk patients accessing the facilities during the strikes. Alternatively, it may also be due to reduction in the numbers of unnecessary or elective surgical procedures, (Caesarian sections) and their attendant risks [1]. The ART clinic also saw a considerable decline in mortality, likely attributable to the large decline in numbers of patients visiting the clinic.

Our results are consistent with multiple studies in industrialized nations [1, 2, 22, 23, 25–27], yet they’re somewhat surprising. Good facility based care have been shown to prevent excess mortality [28]. Nevertheless, there could be iatrogenic risks of hospital stays including hospital acquired infections, incorrect treatment, medical errors and complications from surgical procedures [29–32].

### Macro-mortality impact of strikes

No adverse mortality impact of any of the health professionals’ strike could be demonstrated. It is possible that patients died elsewhere. There is no death registry where macro-mortality in the catchment areas of the four facilities can be compared for trends in the strike and non- strike periods. In a population-based study of mortality impact of strikes, using demographic surveillance data, no change in the mortality during strike vs non-strike periods was noted [33]. Data from strikes in Los Angeles County also saw no population level mortality change [25].

### Mortality as a metric of impact

Mortality is a ‘late-stage’ indicator of health status and may be a poor metric for the full impacts of health worker strikes [1]. It’s possible that the disruption of health services impacts morbidity. Disruption in health services was associated with an increase in reported incidence of Chlamydia infections [34] and an increase in uncontrolled hypertension among known hypertensives on follow up, during a long physician strike [16]. Though we did not demonstrate mortality impacts, further study is needed to determine the morbidity effects of strikes.

#### The role of decongestion

Given that the numbers of patients coming to the public facilities significantly declined during the strike period, it is possible that the strike related mortality decline represents a correction in workload for facilities and staff, namely decongestion with possible benefits in the increased quality of care. The consistent decline in mortality in every department supports this. The converse finding in AICKH, further upholds this argument. The physicians’ strike stretched the hospital’s capacity and cite that increased mortality rates might be related to overstretched health workers with an attendant decline in quality of care as one possible explanation for the findings [35]. AICKH was an alternative facility accessible to many patients who might utilize a public hospital due to the relatively low user fees.

Thus, possibly in addition to fewer mortalities due to elective surgery, the decongestion of public facilities during strikes may account for the decreased mortality during strikes.

### Patient dumping

KNH had the highest mortality at baseline and thereafter. It’s the region’s most specialized public facility and largest teaching and referral hospital and likely receives the more severely ill patients. This may explain the significantly higher baseline mortality. Due to its geographical location, proximal to several large private hospitals it may also be much more subject to patient dumping, defined as transfer of patients from private facilities who are considered undesirable usually for financial reasons. These patients tend to be poor and seriously ill [36]. During the strike, fewer patients may have been transferred, contributing to the large decline in mortality.

## Strengths & Limitations

To our knowledge this is the first study that has quantified the mortality impacts of multiple cadres of health workers, across several departments in a number of facilities in Kenya. Most of the mortality records in the sampled facilities were complete, therefore the study findings are less subject to the biases of missing data.

There were several limitations. As this was an ecological study, it was not possible to discern whether patients who came in during the strike were different in any way from the non-strike period. If these patients had less serious morbidity, then that could explain decreased mortality during the strike. This would be quite unusual, since most patients cannot afford private health care, they’re likely to keep away for trivial ailments during a strike. Some studies have shown no differences in the morbidity level of patients during the strike versus the non-strike period [25]. Further, such patients would likely have presented in the post-strike period with more severe morbidity. However, we observed no increase in mortality in the post-strike period, although the time period observed was shorter than previous intervals.

There were challenges obtaining information on the numbers and cadres of health personnel in the various departments for the period under study. Hence, we cannot speculate on workload or quality of care.

In previous studies, the mortality decline is speculatively attributed to a delay in recording deaths during the strike period or a low level of curtailment of services [1]. In all the facilities we sampled, month to month aggregate patient data were available in every department for the defined period.

Theoretically, the preservation of emergency services during the strike period could have contributed to the decline in mortality by stabilizing acutely ill patients increasing their survival after admission. The decline in the numbers of patients attending the facilities during the strike might have further improved quality of care. However, the study did not by design collect data on the severity of presenting illnesses, the numbers of providers who continued to work, or quality of care in the respective periods. Therefore, we cannot definitively conclude this. Furthermore, it has been shown in our settings the vast majority of the deaths occur outside a health facility, therefore a strike by curative health services might have less of an impact if a large proportion of the population are not attending hospital for treatment [33].

## Conclusion

We recognize quantifying the health impacts of interventions, or absence thereof is often challenging. Interventions occur in complex environments and may require a long duration of observation to detect changes [34]. Nevertheless, we observed declines in facility based mortality during health worker strikes’. The decline is possibly associated with reduced patient volumes, we surmise that decongestion may have contributed. In a country where 80% of the Kenyans lack financial protection from health care costs, Judicious investment in the health infrastructure and staffing may decrease congestion and improve quality of care with possible mortality decline.

## Data Availability

The datasets used and/or analysed during the current study are available from the corresponding author on reasonable request.

## Abbreviation

AICKH: AIC Kijabe Hospital
ART: Anti-Retroviral Treatment Clinic
CO: Clinical Officer
IRB: Institutional Review Board
IRG: Internal Research Grant
KEMRI: Kenya Medical Research Institution
KNH: Kenyatta National Hospital
MH: Mbagathi Hospital
MO: Medical Officer
SH: Siaya Hospital
SERU: Scientific Ethics and Review Unit
US: United States
